# Impacts of management practices on habitat selection during juvenile mountain lion dispersal

**DOI:** 10.1002/ece3.70097

**Published:** 2024-08-01

**Authors:** John F. Randolph, Julie K. Young, David C. Stoner, David K. Garcelon

**Affiliations:** ^1^ Department of Wildland Resources Utah State University Logan Utah USA; ^2^ Ecology Center Utah State University Logan Utah USA; ^3^ Institute for Wildlife Studies Arcata California USA

**Keywords:** connectivity, cougar, human‐wildlife conflict, movement ecology

## Abstract

Dispersal is a complex series of movements before an individual establishes a home range. Animals must travel and forage in unfamiliar landscapes that include anthropogenic risks such as road crossings, harvest, and urban landscapes. We compare dispersal behavior of juvenile mountain lions (*Puma concolor*) from two geographically distinct populations in California and Nevada, USA. These two sites are ecologically similar but have different management practices; hunting is permitted in Nevada, whereas mountain lions are protected in California. We used GPS‐collar data and net‐squared displacement analysis to identify three dispersal states: exploratory, departure, and transient home range. We then compared each dispersal state of the two mountain lion populations using an integrated step selection analysis (iSSA). The model included explanatory variables hypothesized to influence one or more dispersal states, including distance to forest, shrub, water, hay and crop, developed lands, and four‐wheel drive roads, as well as elevation and terrain ruggedness. Results revealed consistent habitat selection between sites across most landscape variables, with one notable exception: anthropogenic covariates, including distance to developed land, distance to hay and crop, and distance to four‐wheeled drive roads, were only statistically significant on modeled habitat selection during dispersal in the population subject to hunting (i.e., Nevada). Results suggest that hunting (pursuit with hounds resulting in harvest) and non‐lethal pursuit (pursuit with hounds but no harvest allowed) increase avoidance of anthropogenic landscapes during dispersal for juvenile mountain lions. By comparing populations, we provided valuable insights into the role of management in shaping dispersal behavior.

## Graphical abstract

We compare dispersal behavior of juvenile mountain lions (*Puma concolor*) from two geographically distinct populations in California and Nevada, USA. Mountain lions selected anthropogenic landscape features differently, suggesting that hunting and non‐lethal pursuit increase avoidance of anthropogenic landscapes during dispersal for juvenile mountain lions.

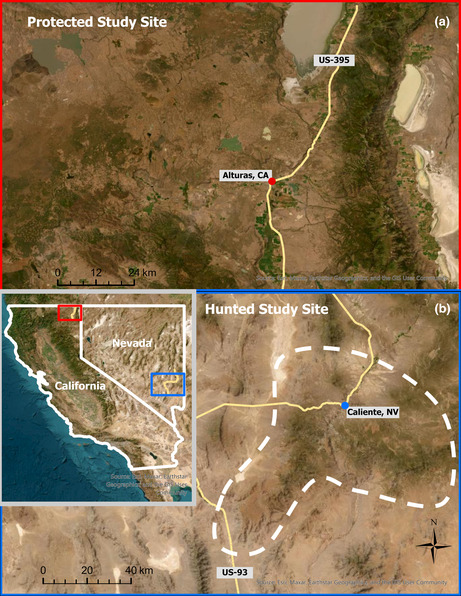

## 1 INTRODUCTION

Dispersal is the movement of an animal from its natal range to the place where it reproduces if it survives (Howard, 1960) and is a central component of an individual's fitness. Benefits from dispersal include reduced competition for resources and improved reproductive success (e.g., finding suitable mates and reduced inbreeding depression; Oliveira et al., 2022). Dispersal also facilitates demographic and genetic connectivity within metapopulations, benefiting individuals and populations (Lowe & Allendorf, 2010).

Despite the benefits of dispersal, it also poses considerable risks (Bonte et al., 2012). During dispersal, individuals navigate unfamiliar and lower‐quality habitats in search of vacancies to establish home ranges (Anderson et al., 2004; Huck et al., 2010). Traveling through fragmented and unfamiliar terrain increases vulnerability to intraspecific strife, predation, human conflict, and human‐related mortality, including vehicle collisions, depredation, and harvest pressure (Andrén et al., 2006; Johnson et al., 2010; Riley et al., 2014; Soulsbury et al., 2008). While navigating inferior or marginal habitat, dispersing juveniles also face energetic strain from a lack of foraging opportunities or poor success rates (Benoit et al., 2020; Palomares et al., 2000; Smith, 1993), making the process risky.

Dispersal can be facilitated or impeded by the degree of landscape connectivity (Taylor et al., 1993). Reductions in connectivity stemming from habitat loss and fragmentation, often caused by anthropogenic development and use, are problematic for juvenile dispersal. Yet metapopulation studies have improved our understanding of the impacts of fragmentation on wide‐ranging species and shown that juvenile dispersal is a critical link connecting fragmented subpopulations (Anderson et al., 2004). Large carnivores, for example, require large home ranges and can often travel long distances daily (Gittleman & Harvey, 1982). Organisms with these traits suffer most from habitat loss and fragmentation due to low population densities and high edge‐area ratios that bring them into contact with anthropogenic landscapes, and consequently with humans. Encounters with anthropogenic landscapes may elevate the risk of human‐related mortality for large carnivores (Naude et al., 2020; Woodroffe & Ginsberg, 1998). Decreased connectivity can directly impact fitness by constraining juvenile dispersal and indirectly affect genetic diversity, potentially leading to inbreeding depression (Crooks, 2002; Heim et al., 2019; Pelletier et al., 2012; Riley et al., 2014), or local extirpations (Benson et al., 2019).

Mountain lions (*Puma concolor*) are large‐bodied, obligate carnivores found throughout the Americas. Because of their large body size and high trophic level, they commonly occur at low densities, exhibit large home ranges, lack a distinct mating season, and rely mainly on immigration as a source of recruitment (Hemker et al., 1984; Lindstedt et al., 1986; Logan et al., 1986; Logan & Sweanor, 2001; Robinette et al., 1961). They can raise young year‐round with a natal period that typically spans 13–17 months before juveniles disperse (Jansen & Jenks, 2012). Upon reaching independence, approximately 50% of juvenile females exhibit philopatry (establishment of an adult home range near or overlapping their natal range; Stoner et al., 2013), whereas the majority of males disperse, and travel significantly farther from their natal home range than dispersing females (Choate et al., 2018; Sweanor et al., 2000; Thompson & Jenks, 2010). This behavior is driven by territorial intolerance of juvenile males by adult males already living in the natal range, prompting juvenile males to disperse (Sweanor et al., 2000). Newly independent juveniles possess poorly developed hunting skills, which can lead them to seek easily accessible resources, such as livestock, roadkill, or prey in urban areas (Stoner et al., 2021). This period of exploratory, nomadic movements coupled with poor hunting skills, means dispersing juveniles are more likely to encounter human disturbance and anthropogenic barriers than residents (Beier, 1995; Dyke et al., 1986; Riley et al., 2014). Yet, mountain lions are predominantly generalist species capable of surviving across a variety of landscapes, ranging from remote wilderness to more developed areas (Coon et al., 2019), and dispersing juveniles can survive providing they obtain sufficient food, avoid intraspecific strife, navigate the complex gradient of anthropogenic obstacles, and minimize human conflict risk.

Conflict with humans is one of the primary causes of carnivore mortality (Woodroffe & Ginsberg, 1998). Sources of conflict consist primarily of livestock or pet depredation (i.e., retaliatory killing of a mountain lion that killed livestock or a pet; Torres et al., 1996), public safety (i.e., lethal removal of a mountain lion that causes risk to the public; Mattson et al., 2011), or depredation on sensitive wildlife species (Rominger, 2018). The typical management response to these conflicts is the lethal removal of the offending animal. Human‐carnivore conflict is prevalent in areas of expanding urbanization, which disrupts landscape connectivity and degrades suitable habitat (Benson et al., 2023; Stoner et al., 2023; Vickers et al., 2015), and in rural areas where farms house small‐hoofed stock (Mazzolli et al., 2002; Weaver, 1978).

Mountain lions are legally hunted throughout most of their range in the western USA, except for in California. Most of this is conducted by pursuing mountain lions into trees or rocky cliffs with the aid of trained hounds. To accommodate this form of hunting, most Western state agencies offer hunters the opportunity to train their hounds during non‐lethal pursuit seasons. This allows hunters with hounds to track and pursue mountain lions without harvesting. Although the terms hunting and harvest are typically used interchangeably, we define hunting as the pursuit or search for mountain lions, while harvest specifically refers to the lethal take of a mountain lion. There has been an overall increase in juvenile harvest reported across the western United States (Elbroch et al., 2022), which influences recruitment and impacts a population's age structure (Cooley, Wielgus, Koehler, & Maletzke, 2009; Logan & Runge, 2021; Newby et al., 2013; Robinson et al., 2008; Stoner et al., 2006). Harvest pressure and habitat quality have also been shown to influence population dynamics (Andreasen et al., 2012; Lindzey et al., 1992). Harvest can influence post‐dispersal habitat selection; mountain lions dispersing in protected populations establish in lower‐quality habitat while mountain lions dispersing in a harvested population will move to equal‐quality habitat (Stoner et al., 2013). This difference likely reflects density‐dependent habitat selection in protected populations (Fretwell & Lucas, 1969).

Because dispersal directly benefits individual survival, reproductive success, and recruitment, as well as indirectly benefits population genetics and viability, it is crucial to understand how different management practices may affect this life stage (Nisi et al., 2023). Yet, we rarely have fine‐scale habitat selection data to understand how differing anthropogenic pressures influence dispersal behavior. Our goal was to assess fine‐scale habitat selection during juvenile dispersal in two mountain lion populations subjected to contrasting management regimes and levels of anthropogenic land uses. We hypothesized that the hunted population would avoid anthropogenic features, but the protected population would be indifferent to these same features as they would not associate them with mortality risk (Smith et al., 2015; Suraci et al., 2019). By comparing two populations subjected to differing management practices, we aim to understand the effects of anthropogenic pressure on juvenile dispersal and shed light on the impacts of hunting and non‐lethal management practices (non‐lethal pursuit seasons) on animal behavior, as well as landscape and population connectivity.

## 2 MATERIALS AND METHODS

### 2.1 Study area

**FIGURE 1:**
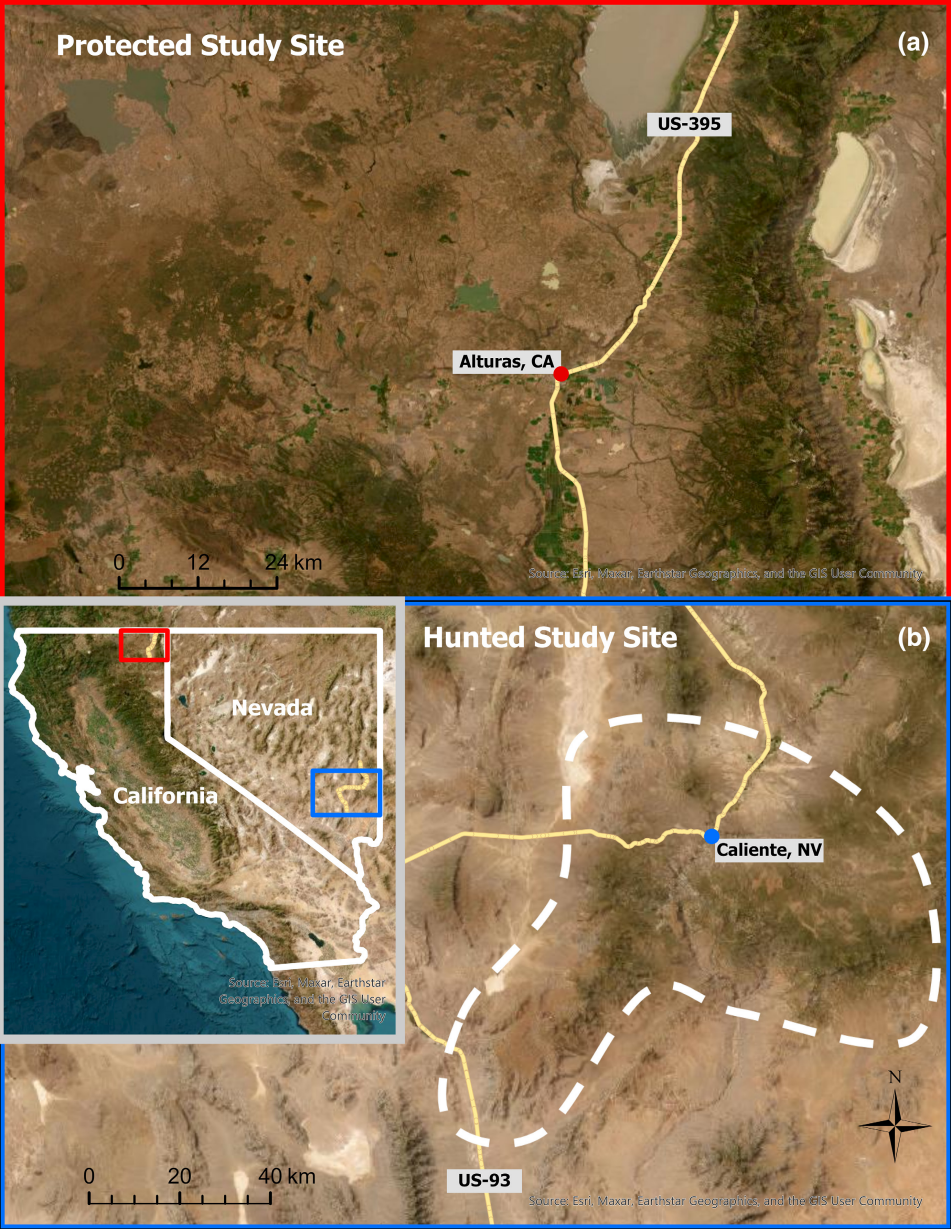
Maps of (a) the Modoc County, California, USA, protected site and (b) a section of Lincoln County, Nevada, USA, featuring the hunted site outlined by a white dashed polygon.

We conducted this study in two sites within the Great Basin ecoregion of the western United States—one in northeastern California (hereafter, the protected site) and the second in southeastern Nevada (hereafter, the hunted site; Figure 1). While both populations are subject to lethal removal for depredation, only the hunted site is also subject to recreational hunting and harvest. The protected site was in Modoc County, California, on the Modoc Plateau and covered 10,890 km^2^ (lat: 41.49450, long: −120.54262). The region experiences temperatures ranging from −11°C in the winter months to 32°C in the summer (Riegel et al., 2006). Elevations vary from 1219 to 2973 m across the county. Annual precipitation can vary, with a range between 17.8 and 121.9 cm (Daly et al., 1994). The dominant vegetation in the area was sage steppe, juniper (*Juniperus occidentalis*) woodlands, conifer forest, and agriculture (Riegel et al., 2006). In higher‐elevation habitats, the vegetation is predominantly ponderosa pine (*Pinus ponderosa*) and Jeffery pine (*Pinus jeffreyi*), transitioning into juniper and sagebrush steppe habitats within the plateaus. Located at the center of the county is Alturas, California, a small town with a population of 2658. Landownership across the plateau was primarily federal and state lands (US Forest Service Modoc National Forest, Bureau of Land Management, U.S. Fish and Wildlife), interspersed with private lands. Primary mountain lion prey consisted of mule deer (*Odocoileus hemionus*), feral horse (*Equus caballus*), pronghorn (*Antilocapra americana*), coyote (*Canis latrans*), and beaver (*Castor canadensis*). Mountain lions are the apex carnivore inhabiting the protected site, with black bears (*Ursus americanus*) present in some portions of the site. Mountain lion hunting was banned in California in 1972, and in 1990 they became a protected species under the California Wildlife Protection Act. Nevertheless, mountain lions are still lethally removed through the issuance of depredation permits in response to verified cases of predation on livestock or for public safety. In 2017, California implemented a three‐strike process to reduce the number of lethal permits issued for depredations. Between 2018 and 2022, 15 mountain lions were removed from the protected site (0.01 mountain lion depredation/100 km^2^/year; California Department of Fish and Wildlife, Unpublished data).

The hunted site was in the Delamar and Clover Mountain ranges within Lincoln County, Nevada, and covered ~4995 km^2^. Elevations vary from 1371 to 2449 m in the Delamar and Clover ranges. The site experiences annual mean precipitation ranging between 10.6 and 40.3 cm, and average temperatures fluctuate from 5.2 to 22.5°C (PRISM Climate Group, 2023). The most common vegetation types were semi‐arid pinyon‐juniper (*Pinus monophylla*, *Juniperus osteosperma*) woodlands and sagebrush steppe. Near the center of this site lies Caliente, Nevada, a small town with a population of 1009. The Bureau of Land Management primarily managed these ranges with minimal private and local municipal land ownership. The mountain lion prey base was similar among sites, consisting of mule deer, feral horses, desert bighorn sheep (*Ovis canadensis*), and pronghorn. Mountain lions were the apex predator, and bears were not present. Mountain lions in this site can be hunted year‐round with no more than two lions harvested per person per year using hounds or opportunistically. The use of hounds is more frequent during the winter months when persistent snow cover facilitates tracking. Harvesting mountain lions through trapping is illegal. From 2018 to 2022, 27 mountain lions were harvested in the study site (0.05 mountain lion harvest/100 km^2^/year; Game Management Units 241, 242, 243, and 223), and one mountain lion was removed due to livestock depredation (0.0002 mountain lion depredation/100 km^2^/year), giving a total of 28 individuals removed from the hunted population (0.06 mountain lion removals/100 km^2^/year; Nevada Department of Wildlife, Unpublished data).

### 2.2 Capture and collaring

From 2016 to 2022, mountain lions in the protected site were captured using cage traps and occasionally hounds (Ewanyk, 2020). All animals were fitted with GPS collars (Vectronic, Lotek, and Sirtrack), programmed at a 1‐ or 2‐h fix rates that uploaded approximately every other day. GPS collars were fitted on dispersal‐age juveniles (13–24 months; Beier, 1995; Cooley, Wielgus, Koehler, Robinson, & Maletzke, 2009), each equipped with a drop‐off mechanism. The drop‐off mechanism was programmed based on the age of the juvenile at the time of capture and ranged from 8 months for juveniles that were still growing to 2 years for juveniles that were close to adult size. Animal handling was approved by two Institutional Animal Care and Use Committees (UC Davis protocol #22408 and USU protocol #12972).

All data from the hunted site were collected between 2018 and 2021 and provided by the Nevada Department of Wildlife (NDOW) for this study. Mountain lion captures began in the Delamar Mountains as part of a desert bighorn sheep study in 2018, with capture efforts expanding into the Clover Mountains in 2020. Hounds and foot snares were used to opportunistically capture and collar mountain lions following methods by Jansen and Jenks (2012). Mountain lions were fitted with GPS collars (Vectronic) programmed at a four‐hour fix rate. Capture methods and handling followed guidelines from the American Society of Mammologists (Sikes & Gannon, 2011), under approval from an NDOW veterinarian.

### 2.3 Data analysis

#### 2.3.1 Movement identification and characterization

Since some juveniles were captured with their mothers while others were already independent, we considered all juveniles independent at the start of a dispersal event. To delineate differing movement states for dispersing juveniles, we used net square displacement (Bunnefeld et al., 2011), using one GPS location per day for each individual in the net‐squared displacement plot. We then used the definitions from Bunnefeld et al. (2011) to identify three distinct movement states: exploratory, departure, and transient home range (defined in Table 1). After identifying each movement state, we removed a three‐day transition period from the beginning of the state and created a new step burst. Juvenile mountain lions were collared as both dependent (with mother) and independent (without mother); we considered all dependent juveniles to be within their natal home range. For independent juveniles whose birthplace was uncertain, we classified home‐ranging behavior around the capture site for periods longer than a month as their natal home range, similar to Karelus et al. (2021) (Bunnefeld et al., 2011). Exploratory behavior occurs when the animal leaves and then returns to its natal range, typically depicted as a long step length travel, while transient home range behavior involves attempts to establish a new range that is ultimately abandoned (centralized short step lengths; Beier, 1995). For both behaviors, a sub‐adult/adult home range is not established. Departure represents instances where the animal leaves its natal range and does not return. We estimated when individuals shifted between these states (Bunnefeld et al., 2011) using R package AMT (Signer et al., 2019; Table 1). Depending on the number of dispersal behaviors identified, we included one or more movement states for each individual in the subsequent habitat selection analysis.

**TABLE 1:** Definitions of the three dispersal behavior states from Bunnefeld et al. (2011) to categorize step data obtained from GPS‐collars on juvenile mountain lions in a protected (Modoc, California, USA) and hunted population (Lincoln, Nevada, USA).

Behavioral state	Definition	Net‐squared displacement segmenting
Exploratory	Departure from natal range but later returns	Nomadic movement away from the natal home range but ultimately returns. Similar to a migration net‐squared displacement plot but on a compressed time scale
Departure	Departure from natal range without any return	Departure from the natal home range in search of establishing an adult home range. This is depicted in the dispersal net‐squared displacement plot
Transient home range	Home‐ranging behavior to explore the quality of habitat	Nomadic movement from natal home range and displays the home range net‐squared displacement plot before later abandoning that range. This is depicted in the mixed net‐squared displacement plot. If the collar dropped when displaying home‐ranging behavior, we classified it as a transient home range if data were obtained for <6 months and as an established range if data were obtained for >6 months

#### 2.3.2 Integrated step selection analysis

We examined juvenile mountain lion dispersal and habitat selection using integrated step selection analysis (iSSA; Avgar et al., 2016). The iSSA uses straight line segments between two consecutive locations (start and end), hereafter referred to as steps, as the unit of observation. We analyzed habitat features at the start of each movement segment to understand how covariates influence movement characteristics, specifically examining step length (the distance between two GPS points) and turning angle (the change in trajectory from the second to third GPS point). We used habitat features associated with the end location to examine habitat selection by the individual. To account for different sampling rates between sites, we resampled GPS locations of mountain lions in the protected site to four‐hour fix rates to match the hunted site. We used a ±10‐min window from the fix rate to account for missed or delayed fixes. If two locations were not within the 10‐min window of the fix rate, they were not considered consecutive locations and were excluded. We then removed non‐movement data such as kill‐site GPS clusters using rASF in Program R (Mahoney & Young, 2017; R Core Team, 2022, version 4.2.2) to avoid selection bias during non‐movement states. Our cluster identification parameters included a minimum fix count of four locations, a spatial buffer of 150 m, and a temporal buffer of 24 h. We kept the first GPS point of an identified cluster as the conclusion of the incoming step and the final GPS point to commence our departure step from the identified cluster. To generate random steps, we created a site‐specific step length distribution and turning angle distribution for each movement state. We then generated 20 random steps based on these distributions for each GPS location to compare available and used steps (Nisi et al., 2022).

We considered the influence of various selection and movement covariates identified in previous mountain lion habitat studies (Benson et al., 2023; Dellinger et al., 2020; Gigliotti et al., 2019; Nicholson et al., 2014; Robinson et al., 2015), and after conducting a correlation analysis on these covariates, we then removed one variable from each pair with correlation coefficients exceeding .60. The covariates analyzed included topography (terrain ruggedness index and elevation; Table 2), distance to anthropogenic features (roads, agriculture, and structures; Table 2), and distance to select land cover types (shrub, forest, and water; Table 2). We also calculated the log of all distance‐to variables to allow more sensitivity to distances closer to that land cover (Ladle et al., 2019; Nisi et al., 2022). All distance‐to variables in the global model and results are log‐transformed. We reformatted coordinate reference systems and resampled raster pixels to 30 × 30 m using ArcGIS Pro V. 3.1.1 (ESRI, 2023).

**TABLE 2:** Overview of variables source data for selected covariates in the integrated step selection analysis to compare dispersal movement of juvenile mountain lions from protected and hunted populations. All units were in meters.

Variable	Definition	Resource
Distance to developed landcover	Open space, low intensity, medium intensity, high intensity	National Land Cover Database 2021; Dewitz (2023)
Distance to hay and crop		National Land Cover Database 2021; Dewitz (2023)
Distance to forest	Evergreen, mixed, deciduous	National Land Cover Database 2021; Dewitz (2023)
Distance to shrub	Grassland, herbaceous	National Land Cover Database 2021; Dewitz (2023)
Distance to water	Open water, emergent herbaceous wetlands, woody wetlands, linear streams, and rivers	National Land Cover Database 2021; Dewitz (2023) and United States Geographical Survey National Hydrography Dataset (2023)
Distance to four‐wheeled drive roads		United States Geographical Survey National Transportation Dataset (2023)
Elevation		Elevatr R Package; Hollister et al. (2017)
Terrain Ruggedness Index		Elevatr R Package; Hollister et al. (2017)

We extracted habitat covariates at all used and available steps and fit a global step selection model for each of the three dispersal behavioral states with program R (R Core Team, 2022, version 4.2.2) package AMT (Signer et al., 2019) to estimate selection of habitat variables for each individual (Table 2). Because our study is exploratory in scope, we only examined the global model, which included all variables we hypothesized to influence mountain lion movements and habitat selection (Table 2). We considered interactions between step length and turning angle with all anthropogenic covariates. To obtain population‐level parameters, we used each individual's beta estimate to calculate an inverse‐variance weighted mean for each study site. This provided a log‐relative selection strength (log‐RSS; Avgar et al., 2017) for each covariate by each population.

## 3 RESULTS

### 3.1 Capture and collaring

We captured and fitted GPS collars on 13 juvenile mountain lions (2 females and 11 males) in the protected site. Of these, five males and one female were captured within their maternal range, whereas the others were independent at the time of capture (Table A1 in Appendix 1). There were two mortalities; one died of starvation (1 male), and one was lethally removed for depredation (1 male; Table A1 in Appendix 1). GPS collars provided an average of 298 days (SE ±46 days) of data per juvenile in the protected site. On the hunted site, 12 juveniles (7 females and 5 males) were captured and fitted with GPS collars. Of these, seven were within their maternal home range (3 males and 4 females), one female was already independent, and four were of unknown status (1 male, 3 females; Table A1 in Appendix 1). We recorded eight mortalities; four were harvested (2 females, 2 males), one was removed for depredation (1 female), and three died of unknown causes (2 females, 1 male; Table A1 in Appendix 1). The average duration of data collected from GPS collars in our hunted site was 631 days (SE ±154 days) per juvenile. All individuals from both sites were included in the analysis from their first independent movement until their final dispersal event or time of death.

### 3.2 Movement identification and characterization

Three juvenile males in the protected site did not display any dispersal behavior (Table 1) and were consequently removed from the study, resulting in a sample size of 10 individuals (2 females, 8 males; Table A1 in Appendix 1). Six individuals displayed exploratory behavior one or more times, averaging 47 days (SE ±14 days) in duration, with an average total distance traveled of 154 km (SE ±48 km; Table A2 in Appendix 1). Nine juveniles exhibited departure behavior between February and June, averaging 50 days (SE ±14 days) in duration and traveling a mean total distance of 188 km (SE ±58 km; Table A2 in Appendix 1). Eight juvenile mountain lions exhibited transient home range behavior, with each juvenile spending an average of 38 days (SE ±5 days) in this behavior (Table A2 in Appendix 1). The average distance traveled from their natal ranges to a transient home range was 52 km (SE ±9 km).

In our hunted site, one juvenile male did not display dispersal behavior and was removed from the analysis (Table 1); 11 juveniles (7 females and 4 males) were retained (Table A1 in Appendix 1). There were six juveniles that exhibited an exploratory state, averaging 66 days (SE ±24 days) with an average total distance traveled of 236 km (SE ±64 km; Table A2 in Appendix 1). Departure was observed for eight juveniles between February and December, lasting an average of 45 days (SE ±8 days) and traveling a mean total distance of 160 km (SE ±32 km; Table A2 in Appendix 1). Six juveniles displayed transient home ranges, averaging 150 days (SE ±45 days) and traveling an average distance of 99 km (SE ±11 km) from their natal range.

### 3.3 Integrated step selection analysis

#### 3.3.1 Exploratory state

**FIGURE 2:**
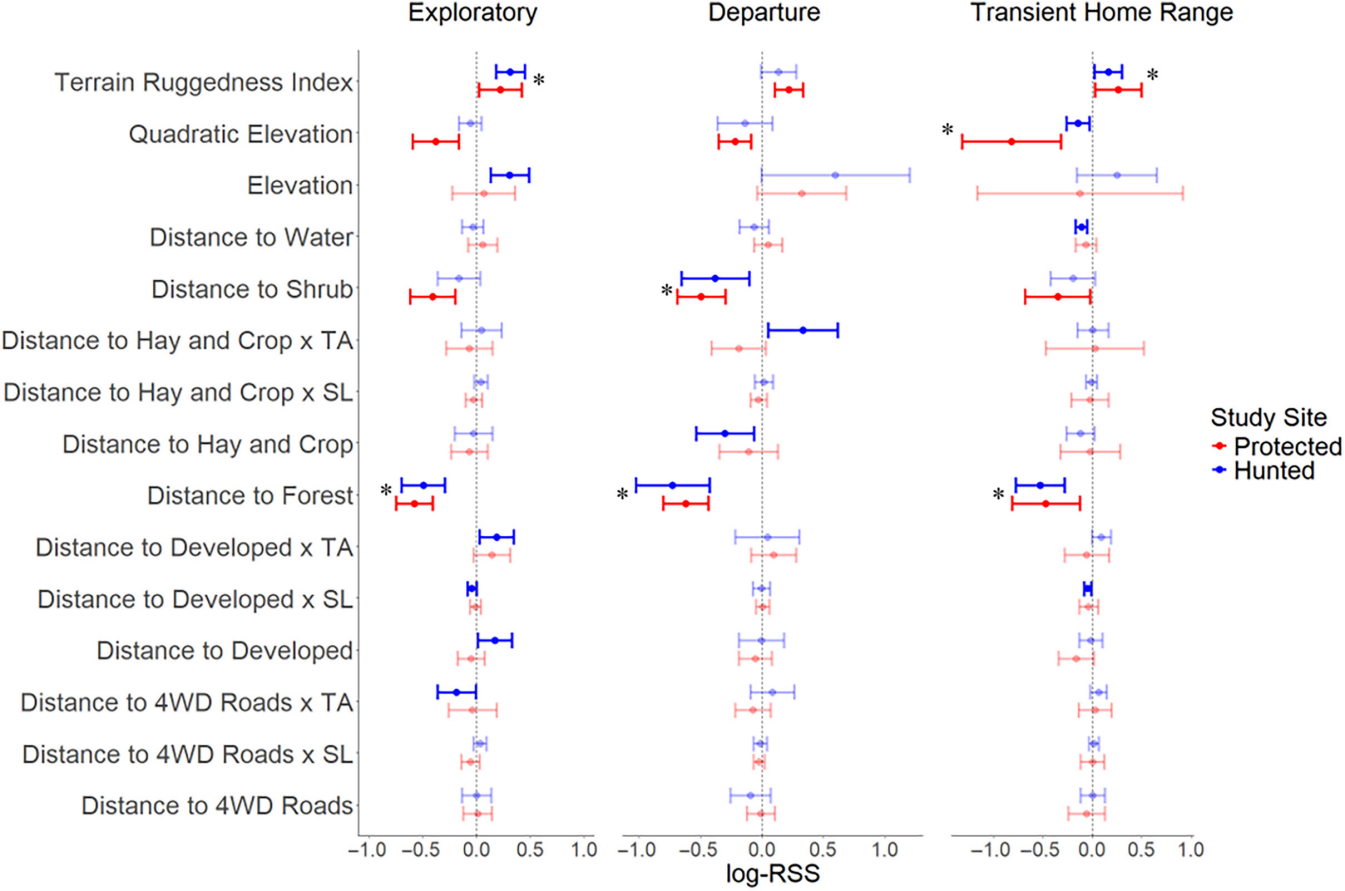
Global model of significant log Relative Selection Strength (log‐RSS), that is, beta coefficient, and 95% confidence intervals for a one‐unit change in the covariate for each dispersal behavior between sites. If a covariate includes an “x”, it indicates an interaction term with either TA (turning angle) or SL (step length). Bold bars represent significant covariates where the estimate and confidence interval do not overlap zero, while faded bars overlap zero and are not considered significant. Covariates where both study sites are significant are marked with an asterisk (*).

We found nine covariates in the global model for the exploratory state that exhibited significance (Figure 2). Among them, six covariates are related to habitat selection, whereas the remaining three were associated with movement. In the exploratory state, mountain lions in both protected (P) and hunted (H) sites selected similarly for forest (P: β = −.582 & H: β = −.496) and terrain ruggedness index (P: β = .223 & H: β = .316; Figure 2). The protected site mountain lions selected distances close to shrub land cover (P: β = −.409), whereas those in the hunted site selected farther distances from developed landscapes (H: β = .169; Figure 2). Mountain lions in the hunted site selected for higher elevations (H: β = .308) while those from the protected site selected for elevations near and around the mean (P: β = −.380; Figure 2). In our hunted site, estimates of step lengths (H: β = −.044) were longer and turning angles were more tortuous in developed landscapes (H: β = .186) and exhibited more direct movements when near or on four‐wheel‐drive roads (H: β = −.186; Figure 2).

#### 3.3.2 Departure state

The global model for the departure state contained six significant covariates (Figure 2). Of these, four were habitat covariates and one was a movement covariate. Mountain lions in both sites selected to be near or within forest (P: β = −.618 & H: β = −.725) and shrub land cover (P: β = −.493 & H: β = −.378; Figure 2). The protected mountain lions selected for higher terrain ruggedness (P: β = .221) and elevation near and around the mean (P: β = −.218; Figure 2). Hunted mountain lions selected for locations near or within hay and crop (H: β = −.299) and turning angles were more tortuous within and near agricultural areas (H: β = .335; Figure 2).

### 3.4 Transient home range state

In the global model for the transient home range state, we identified five significant covariates, which were categorized into four habitat and one movement covariate (Figure 2). Mountain lions in the transient home range state at both sites selected for more rugged terrain (P: β = .264 & H: β = .162), with elevations around the mean (P: β = −.815 & H: β = −.141), and for forest land cover (P: β = −.469 & H: β = −.525; Figure 2). The mountain lions at the protected site selected for shrub habitat (P: β = −.348) and at the hunted site selected for water features (H: β = −.109; Figure 2). Hunted mountain lions had longer step lengths near and within developed landscapes (H: β = −.045; Figure 2).

## 4 DISCUSSION

Mountain lions have the largest latitudinal distribution of any species of wild cat (Kitchener, 1991) and the largest distribution of any wild terrestrial mammal in the western hemisphere (Sunquist & Sunquist, 2002). Where previously studied, juvenile dispersal by mountain lions has been confined to single or neighboring populations (Beier, 1995; Morrison et al., 2015; Newby et al., 2013). Making meaningful comparisons across populations can be difficult due to differences in habitats, weather patterns, and methodologies. By comparing juvenile dispersal behaviors between two populations inhabiting similar basin‐and‐range habitats over the same time period, but with different wildlife management practices, we were able to explore how those management practices may influence movement and habitat selection behaviors. We found minimal differences in habitat selection between our two study sites and across three dispersal states; however, the differences that we found were associated with anthropogenic covariates. As we hypothesized, mountain lions in the hunted site avoided developed landscapes whereas the juveniles dispersing from the protected site did not select for or against developed landscapes.

Due to the challenges in capturing and collaring juvenile mountain lions, we considered some caveats in interpreting our results. Differences we observed may be influenced by varying sex ratios and different age classes (i.e., dependent and independent) of juveniles collared between sites, which also resulted in different numbers of early and late dispersal states between sites. That said, we observed a range of dispersal characteristics within both sites and identified all movement states within both age classes. We also acknowledge that our broad definitions for classifying diverse movements, which exhibit high variability between individuals, may have led to misidentified states. Specifically, our assumption regarding natal ranges of independent individuals, inferred from home‐ranging behavior around the capture site for longer than 1 month, may alternatively reflect a transient home range. Yet these broad definitions enabled us to segment dispersal movements into three states, which allowed us to focus our analysis on similar states. Across the three dispersal states, juveniles selected habitats similar to that used by adult mountain lions in other studies, including forest, shrub, increased terrain ruggedness, and higher elevation (Gigliotti et al., 2019; Nicholson et al., 2014; Robinson et al., 2015). These covariates are also important to herbivores that are the primary prey of mountain lions (Morano et al., 2019; Van Beest et al., 2014) and may facilitate hunting opportunities (Kunkel et al., 1999). As such, our data suggest that dispersing mountain lions predicate habitat selection on the general habitat associations of their primary prey.

The response to anthropogenic covariates differed between the two focal populations. Models of mountain lions in the hunted site indicated habitat selection and avoidance related to anthropogenic factors. During exploratory and transient home range states, we found evidence of avoidance of developed landcover, accompanied by varying movement behaviors. Conversely, during the departure state, there was selection for hay and crop landcover. During the exploratory state, mountain lions in the hunted site exhibited increased step length and more torturous movements observed near or within developed landscapes, potentially driven by perceived risk or hindrance to movement (Dickie et al., 2020). Mountain lions have previously been shown to select areas in proximity to four‐wheel drive and dirt roads for easier movement (Dellinger et al., 2020), suggesting that our observed increased step length could also relate to four‐wheel drive and dirt roads facilitating movement of dispersing mountain lions (Dickie et al., 2020). During the transient home range state, juveniles in the hunted site exhibited straighter movement when near or within developed landscapes. Most studies show mountain lions typically avoid developed landscapes (Riley et al., 2021; Robinson et al., 2015), so it is likely that straight movement (i.e., increased step length) is a behavior exhibited by mountain lions attempting to quickly move past developed areas, areas of high exposure, or those landscapes with little habitat value.

Although juveniles from the hunted population generally avoided developed landscapes, they selected for hay and crop during the departure state. This most likely relates to resource availability (Tucker et al., 2021), as their primary prey species, mule deer, are drawn to agricultural landscapes due to the increased availability and predictability of resources (Anderson et al., 2012). Our study sites experience dramatic seasonal shifts in environmental conditions throughout the year; however, human‐modified agricultural landscapes provide a more predictable and readily available resource for wildlife (Oro et al., 2013; Sih et al., 2011). The selection of hay and crop along with tortuous movements within these habitats suggests that mountain lions could be using these habitats for hunting or scavenging roadkill (Dickie et al., 2020; Stoner et al., 2021). Hay and crop landscapes are typically privately owned and not commonly accessible to hunters, and might also serve as refugia from humans or adult mountain lions (Harden et al., 2005; Proffitt et al., 2013). Established adult mountain lions are also unlikely to regularly use agricultural landscapes (Dickson & Beier, 2002), potentially offering juvenile mountain lions refuge from intraspecific strife (Morrison et al., 2015). Similarly, brown bears (*Ursus arctos*) use anthropogenic landscapes to reduce sexually selected infanticide, as adult males were less inclined to use these habitat types in their home range (Steyaert et al., 2016).

During the exploratory and transient home range states (segment events = 19), we observed avoidance of developed landscapes and altered movements within them. The avoidance observed during the exploratory state may be attributed to juveniles seeking habitat that reflects their natal home range, and therefore maternal preferences (Davis & Stamps, 2004; Riley et al., 2021; Robinson et al., 2015; Stamps & Swaisgood, 2007). They likely transition to using other habitat features as they learn to find areas with increased prey availability, providing more opportunities as they better develop their hunting skills. This is supported by our departure state, wherein dispersing juvenile mountain lions select hay and crop areas. The differences in habitat selection between movement states could suggest that juvenile dispersal is a lengthy learning process.

Developed landscapes represent the most intense form of anthropogenic influence and are often avoided by large carnivores (Boydston et al., 2003; Dickson et al., 2005; Støen et al., 2015). For dispersing juvenile mountain lions, human‐carnivore conflict is unpredictable in time, space, and magnitude, exposing them to risks such as vehicle collisions, public safety concerns, and depredation control (Dellinger et al., 2021; Kertson et al., 2013; Mattson et al., 2011; Thompson et al., 2014). In our study, only mountain lions from the hunted population showed avoidance of developed landscapes, while the protected population did not show selection for or avoidance of any anthropogenic covariates. Most of the developed landscape within the hunted site is situated in and around the town of Caliente, which is completely surrounded by otherwise suitable mountain lion habitat. Additionally, the town attracts ungulates because it is concentrated around perennial water sources. This combination of suitable habitat and increased resource availability could attract dispersing mountain lions. However, our observed response to developed lands might imply that hunting pressure and pursuit cause juvenile mountain lions to avoid this otherwise suitable habitat.

This could suggest a learned avoidance of developed landscapes, potentially influenced by negative interactions with hounds and hunting. Unlike other carnivores that adjust their habitat selection and movement in response to perceived risk during specific hunting seasons (Basille et al., 2013; Lodberg‐Holm et al., 2019; Stillfried et al., 2015), mountain lions in the hunted site consistently avoided developed landscapes during dispersal. The year‐round avoidance behavior observed in hunted mountain lions could stem from several factors. First, it may be attributed to the extended duration of both pursuit and harvest seasons annually, rendering it challenging for the animals to avoid human activity. The presence of hunters and hounds during these seasons could lead individual mountain lions to encounter these threats multiple times throughout the year without being harvested, further reinforcing avoidance behaviors. This avoidance behavior may also be influenced by maternal experience, with young mountain lions learning avoidance tactics from their mothers.

The use of dogs as a tool in wildlife monitoring and management is diverse. Scat detection dogs are employed across the western regions for noninvasive genetic sampling (McKeague et al., 2024; Wasser et al., 2004) and livestock guardian dogs are used to mitigate human‐carnivore conflict through livestock protection (Andelt & Hopper, 2000; Young & Sarmento, 2024). Dogs are also used for hazing nuisance black bears in urban settings (Beckmann et al., 2004). However, the use of dogs for hazing mountain lions has received relatively little scientific attention. Our study found an increased avoidance of developed landscapes by animals exposed to non‐lethal hunting pressure, suggesting mountain lions may select against landscape features correlated with high human activity including areas with dogs. Because hunting and pursuing mountain lions with hounds often occurs in these spaces, pursuit with hounds could provide wildlife managers with a previously underutilized method for reducing human–mountain lion conflicts. However, we can only speculate on the potential impacts with our data. Gathering additional data on specific interactions, including catch‐per‐unit‐effort, sex and age class of animals pursued, hunter encounter rates, and chase distances and return times of mountain lions subjected to pursuit may be a valuable first step in evaluating the efficacy of dogs as a non‐lethal management intervention.

In this study, we leveraged GPS‐collar data from two study sites to compare juvenile dispersal between hunted and protected populations of mountain lions. Harvest of mountain lions is common in most of the western United States and serves multiple purposes, including managing mountain lion populations, mitigating human‐carnivore conflicts, minimizing livestock depredation, reducing predation on ungulate populations, and providing recreational opportunities. However, harvest also influences the success of dispersal and modifies the spatial behavior of harvested species (Logan & Runge, 2021; Newby et al., 2013; Robinson et al., 2008; Smith et al., 2022). Our findings expand our understanding of the influence of hunting on juvenile dispersal movements and habitat selection by mountain lions. We identified similarities in selection with habitat covariates commonly correlated with mountain lions (Gigliotti et al., 2019; Nicholson et al., 2014; Riley et al., 2021; Robinson et al., 2015), except that we found our two sites differed in their response to anthropogenic landscapes. These selection differences highlight the importance for wildlife managers dealing with imperiled populations, habitat loss, and fragmentation to consider the impacts of hunting pressure on dispersing individuals and their recruitment into the population. Wildlife agencies across the western United States should consider how management practices affect both the focal population and the metapopulation. Our results contribute to the growing body of evidence that management practices can have behavioral effects on the movement and habitat selection of juvenile mountain lions during dispersal (Cooley, Wielgus, Koehler, & Maletzke, 2009; Logan & Runge, 2021; Newby et al., 2013; Robinson et al., 2008).

## AUTHOR CONTRIBUTIONS

**John F. Randolph:** Conceptualization (equal); data curation (lead); formal analysis (lead); writing – original draft (lead); writing – review and editing (equal). **Julie K. Young:** Conceptualization (equal); formal analysis (supporting); resources (equal); supervision (lead); writing – original draft (supporting); writing – review and editing (equal). **David C. Stoner:** Conceptualization (equal); resources (equal); writing – original draft (supporting); writing – review and editing (equal). **David K. Garcelon:** Conceptualization (equal); funding acquisition (lead); resources (equal); writing – review and editing (equal).

## CONFLICT OF INTEREST STATEMENT

None declared.

## Data Availability

Data from California can be accessed on Dryad: https://doi.org/10.5061/dryad.hdr7sqvrw. Private Peer Review link: https://datadryad.org/stash/share/jRF6ssHTUggmVCYSrUHAg_7YYoGq60bhY3twxzDjpBQ. Nevada mountain lions are a protected game species under Nevada Administrative Code (NAC) 502.370. As such, raw location data of mountain lions are considered proprietary and cannot be released without written permission from the Nevada Department of Wildlife. Data inquiries can be addressed to Patrick Jackson [pjackson@ndow.org].

## APPENDIX 1

**TABLE A1:** Detailed information on each juvenile mountain lion fitted with a GPS collar, including ID, sex, age, total monitoring duration, and duration within each behavioral state (exploratory, departure, and transient home range).

Study site	Lion ID	Sex	Age	Days collared	Cause of mortality	Exploratory events	Total No. of days	Departure events	Total No. of days	Transient home range events	Total No. of days
Protected	M168	Male	15 months	386	–	–	–	1	141	1	57
	M176*	Male	16 months	109	Starvation	2	18	1	22	1	21
	M197*	Male	19 months	365	–	–	–	1	48	–	–
	M198	Male	12 months	300	–	1	35	–	–	1	41
	M200	Male	17 months	365	–	2	112	1	69	–	–
	M202*	Male	15 months	108	Depredation	–	–	1	11	1	34
	F206*	Female	14 months	176	–	1	17	1	18	1	55
	M208	Male	22 months	299	–	1	46	1	15	1	22
	M281*	Male	19 months	205	–	1	52	1	92	2	36
	F286	Female	16 months	224	–	–	–	1	30	1	39
	M341	Male	18 months	223	–	Did not display any dispersal behavior					
	M282	Male	19 months	223	–	Did not display any dispersal behavior					
	M280*	Male	16 months	224	–	Did not display any dispersal behavior					
Hunted	DF06*	Female	Sub‐adult	1146	–	1	23	1	44	3	421
	DF07	Female	Sub‐adult	633	Harvested	2	91	–	–	–	–
	DF08*	Female	Sub‐adult	2232	–	1	172	1	62	5	234
	DF10	Female	Sub‐adult	82	Depredation	1	32	–	–	–	–
	DM12*	Male	Sub‐adult	547	Harvested	–	–	1	11	–	–
	DF13*	Female	Sub‐adult	939	Unknown	1	60	–	–	1	53
	DF20	Female	Sub‐adult	154	Unknown	–	–	1	46	1	57
	DM21*	Male	Sub‐adult	555	Harvested	–	–	1	30	–	–
	DF24*	Female	Sub‐adult	403	Harvested	1	18	1	76	1	104
	DM33	Male	Sub‐adult	116	Unknown	–	–	1	24	1	30
	DM35*	Male	Sub‐adult	1319	–	–	–	1	68	–	–
	DM17	Male	Sub‐adult	115	–	Did not display any dispersal behavior					

*Note*: Mountain lions with an asterisk (*) by their ID were captured in their mother's home range.

**TABLE A2:** Data on the behavioral states of each collared mountain lion, including the number of days within each state and the total distance traveled (km) for exploratory, departure, and transient home range states.

Study site	Lion ID	Exploratory state			Departure state			Transient home range state		
		Events	Total number of days	Total distance (km)	Events	Total number of days	Total distance (km)	Events	Total number of days	Total distance (km)
Protected	M168	–	–	–	1	141	403.64	1	57	328.32
	M176	2	18	46.36	1	22	24.06	1	21	44.47
	M197	–	–	–	1	48	174.43	–	–	–
	M198	1	35	39.92	–	–	–	1	41	48.33
	M200	2	112	222.87	1	69	273.67	–	–	–
	M202	–	–	–	1	11	35.87	1	34	93.01
	F206	1	17	61.03	1	18	32.48	1	55	116.26
	M208	1	46	308.14	1	15	90.17	1	22	133.87
	M281	1	52	244.66	1	92	506.3	2	36	116.42
	F286	–	–	–	1	30	149.79	1	39	132.34
	M280	Did not display any dispersal behavior								
	M282	Did not display any dispersal behavior								
	M341	Did not display any dispersal behavior								
Hunted	DF06	1	23	127.1	1	44	216.53	3	421	1757.1
	DF07	2	91	482.7	–	–	–	–	–	–
	DF08	1	172	254.87	1	62	122.81	5	234	477.64
	DF10	1	32	133.13	–	–	–	–	–	–
	DM12	–	–	–	1	11	101.18	–	–	–
	DF13	1	60	–	–	–	–	1	53	106.17
	DF20	–	–	–	1	46	146.38	1	57	128.11
	DM21	–	–	–	1	30	117.82	–	–	–
	DF24	1	18	75.21	1	76	323.47	1	104	180.59
	DM33	–	–	–	1	24	35.03	1	30	57.18
	DM35	–	–	–	1	68	215.19	–	–	–
	DM17	Did not display any dispersal behavior								
